# The effects of a canalplasty and a canal wall reconstruction on perceived sound quality: preliminary results

**DOI:** 10.1007/s00405-016-3910-z

**Published:** 2016-02-26

**Authors:** E. van Spronsen, P. Brienesse, F. A. Ebbens, W. A. Dreschler

**Affiliations:** Department of Otorhinolaryngology Head and Neck Surgery, Academic Medical Centre, Meibergdreef 9, 1105 AZ Amsterdam, The Netherlands

**Keywords:** Ear canal, Acoustics, Sound quality

## Abstract

Objective of this work was to evaluate the perceptual effect of the acoustic properties before and after canalplasty and a reconstruction of the posterior canal wall in revision modified radical cavity surgery. This is a prospective study. Twenty normal hearing subjects were presented six simulated sound conditions representing the acoustic properties of six different ear canals (two normal ears, and two pre- and postoperative conditions). The six different real ear unaided responses of these ear canals were used to filter Dutch sentences, resulting in six simulated sound conditions. A seventh unfiltered ‘reference’ condition was used for comparison. Sound quality was evaluated using a seven-point paired comparison rating and a visual analogue scale (VAS). Significant differences in sound quality were found between all conditions and the pre-operative cavity condition (all *p* < 0.001) using both the paired comparison rating and VAS. No significant differences in VAS were found comparing the other conditions with each other. But when using the paired comparison rating, the post-operative canalplasty condition and both the pre and post-operative cavity conditions differed significantly from the other conditions. This explorative study shows that altering the acoustics of the OEAC after a canalplasty and a reconstruction of the ear canal in revision modified radical cavity surgery results in perceivable changes in sound quality. It is likely that these changes are primarily due to volume changes. To which extent these changes are of clinical importance remains to be determined.

**Electronic supplementary material:**

The online version of this article (doi:10.1007/s00405-016-3910-z) contains supplementary material, which is available to authorized users.

## Introduction

It is well known that the external auditory ear canal plays a role in the transfer of sound from the concha to the tympanic membrane. It acts as a resonant tube [1]. Several manuscripts have reported a change in resonance acoustics when the osseous external auditory canal (OEAC) is modified surgically [2–5]. Our group frequently encountered patients reporting a post-operative improvement or deterioration of sound quality while pure tone audiometry (PTA) showed no relevant changes at all. This could be explained by the abovementioned literature findings. Surgical alteration of the OEAC was shown to alter the resonant frequency substantially and the peak amplitude significantly [2, 6]. An evaluation of the perceptual consequences of the largest surgical alteration (drilling a modified radical cavity) has shown significant effects [6] on perceived sound quality. Although some questions still remain regarding possible habituation and its relevance in regular clinical care, these effects cannot be disregarded. Besides drilling a modified radical cavity, various other surgical alterations of the OAEC can be performed. A canalplasty procedure can be performed to widen the OAEC, to create a self-cleansing and patent external auditory canal. Is it possible that such a procedure leads to similar acoustic effects? And, does reconstruction of the posterior canal wall after prior radicalisation, thereby restoring more ‘normal’ dimensions of the OAEC, lead to a significant effect in perceived sound quality?

The purpose of this study was to test whether and to which extent sound quality is affected by surgical changes in the shape of the external auditory canal in one individual patient. For this purpose we compared the acoustic effects pre- and post-operatively in patients undergoing a canalplasty procedure and a revision radical cavity surgery with reconstruction of the posterior canal wall.

## Participants and methods

### Subjects

For the listening experiments, 20 individuals with normal hearing were included. This group was comprised of 14 (70 %) female and 6 (30 %) male participants with an average age of 32.9 year (median 29.0 ranging from 22 to 60.6 years). Their hearing thresholds were 20 dB HL or better at 0.25, 0.5, 1, 2, 4 and 8 kHz. All participants were healthy and had no history of ear disease. All participants underwent otoscopy showing no pathology (except a few cases of minor myringosclerosis). Informed consent was obtained from all individual participants included in the study. IRB was acquired and given by the ethical committee review board to perform this study.

### Sound recordings

Two patients agreed to participate in this study. One patient suffered from chronic external otitis due to extensive exostosis formation. A canalplasty procedure was indicated and performed as was described by our group in an earlier manuscript [7]. Very briefly, this technique uses a skin flap that allows complete circular drilling and limits potential skin loss. No grafts are used as the skin is spared and healing is secondary. Two REURs were obtained, one pre-operatively and one after successful healing. The other patient was indicated for revision modified radical cavity surgery due to a troublesome cavity. During this procedure a partial obliteration using hydroxyl-apatite granules of the mastoid bowl was performed and a new posterior canal wall was reconstructed with cartilage and a midtemporal flap. This procedure was a slight modification of the technique described by Yung et al. [8]. The modification being that the inferiorly based flap is not used in our series as the midtemporal flap alone suffices. Again, two REURs were obtained, one pre-operatively and one after successful healing (this being approximately 3 months post-operatively). Two other (non-participating) volunteers with no history of ear disease and having normal ear canals determined by regular otoscopy agreed to participate as normal controls. In both these volunteers, a REUR was obtained from one ear.

### Simulation of the acoustic properties of six individual ear canals

The acoustic properties of the ear canal can be characterized by measuring the real ear unaided response (REUR) [6]. This response is measured with a probe microphone inserted into the external auditory canal and shows the sound pressure level at the eardrum after the presentation of a well-defined broadband sound stimulus. Differences between individual REURs therefore represent differences in acoustic properties of individual ear canals. For instance, the acoustic effect of an ear canal with a radical cavity can be simulated in a normal ear canal by filtering the incoming sound stimulus using the difference of the REUR of a normal ear and the REUR of a cavity ear. The filtered sound stimuli, presented to a normal ear, should result in the same distribution of sound pressure at the eardrum as in the original radical cavity [6].

**Fig. 1:**
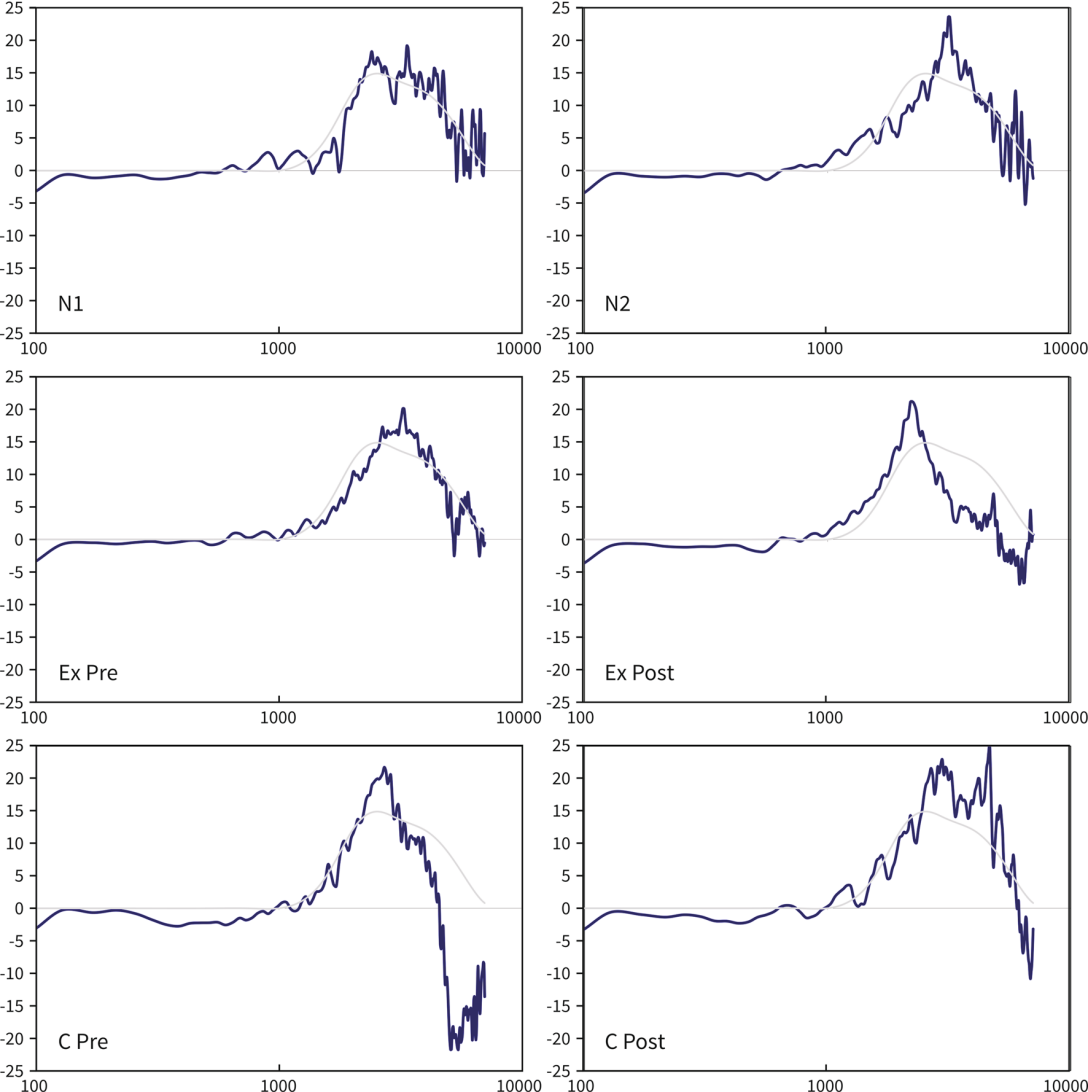
Measured real ear unaided gain (REUG) of all conditions: two ‘normal’ ear canals (N1 and N2), a pre- and postoperative condition of a patient with exostosis who underwent canalplasty (Ex Pre and Ex Post) and a pre- and postoperative condition of a patient with a radical cavity who underwent a revision surgery with cartilage reconstruction of the posterior ear canal (C Pre and C post) (*dark lines*). In each window the average adult REUG is also depicted (Dillon) (*light line*). The REUG data are depicted on the same scale from 100 to 7000 Hz on the frequency *x* axis, and −25 to 25 dB (gain) on the *y* axis

We used Dutch speech recordings (two male and two female speaker sentences based on the VU98 sentence material [9], filtered to simulate the acoustic properties of six ear canal conditions: two normal ear canals, two pre-operative conditions (ear canal with exostosis and radical cavity), and two post-operative conditions (canalplasty and revision radical cavity surgery with reconstruction of the posterior ear canal wall). The REURs of these six conditions were measured using the REM module of the Affinity 2.0 Hearing Aid Analyzer platform (Interacoustics, Denmark). Figure 1 shows the REUR results of the six conditions, presented as a real ear unaided gain (REUG, being the difference between the incoming broadband stimulus and the REUR). Six filters c.q. simulated conditions were built based on the differences between these six REUGs and the average REUG of a normal adult ear canal (see Table 4.6 in Dillon H (8), page 110 [10]). The seventh ‘reference’ condition consisted of the unfiltered speech material. We included sound samples, using English sentences but the same filters, comparable to those who were presented to the participants in the sound files.

### Perceptual evaluation

The perceptual evaluation experiment was performed with a paired comparison category rating between two fragments (‘A’ and ‘B’), according to ITU-T 1996. Participants were asked which fragment sounded more natural using a seven point comparison rating scale. The paired comparison rating results are denoted on a seven point scale from 3 (the simulated ear canal c.q. filtered signal sounds much more natural than the reference c.q. unfiltered condition) to −3 (the reference condition sounds much more natural than the simulated ear canal). A score of 0 means that there is no noticeable difference perceived in naturalness between the two conditions. These fragments were comprised of the six conditions and each filtered condition was compared to the unfiltered reference condition. All conditions were presented with two male and two female speaker sentences and were measured twice: one time with the filtered sentence as ‘A’ and the reference sentence as ‘B’, and vice versa. With these 48 paired comparisons, together with four control comparisons in which the seventh unfiltered condition was compared with itself, a total of 52 paired comparisons were presented in random order.

The paired comparison category rating task was followed by a visual analogue scale (VAS) score task, evaluating the ‘overall’ sound quality of the seven conditions, zero being the worst possible outcome and 100 the best. Again, the seven conditions were presented in random order by playing four different Dutch sentences.

All of the speech material was presented in free field at a level of 65 dB(A), using a loudspeaker in front of the listener (0^°^ angle).

### Statistical analysis

Statistical analysis was conducted using SPSS 16.0.2 (Chicago, IL, USA). Data are expressed as numbers. Mann–Whitney *U* test was performed to check for significant changes from baseline in the VAS scores. ANOVA multivariate analysis was used to determine the effects of subject, condition, and gender of the speaker on the results. The Bonferroni correction was applied to account for multiple comparisons. *p* values of less than 0.05 were considered statistically significant.

## Results

### Paired comparison ratings

**Fig. 2:**
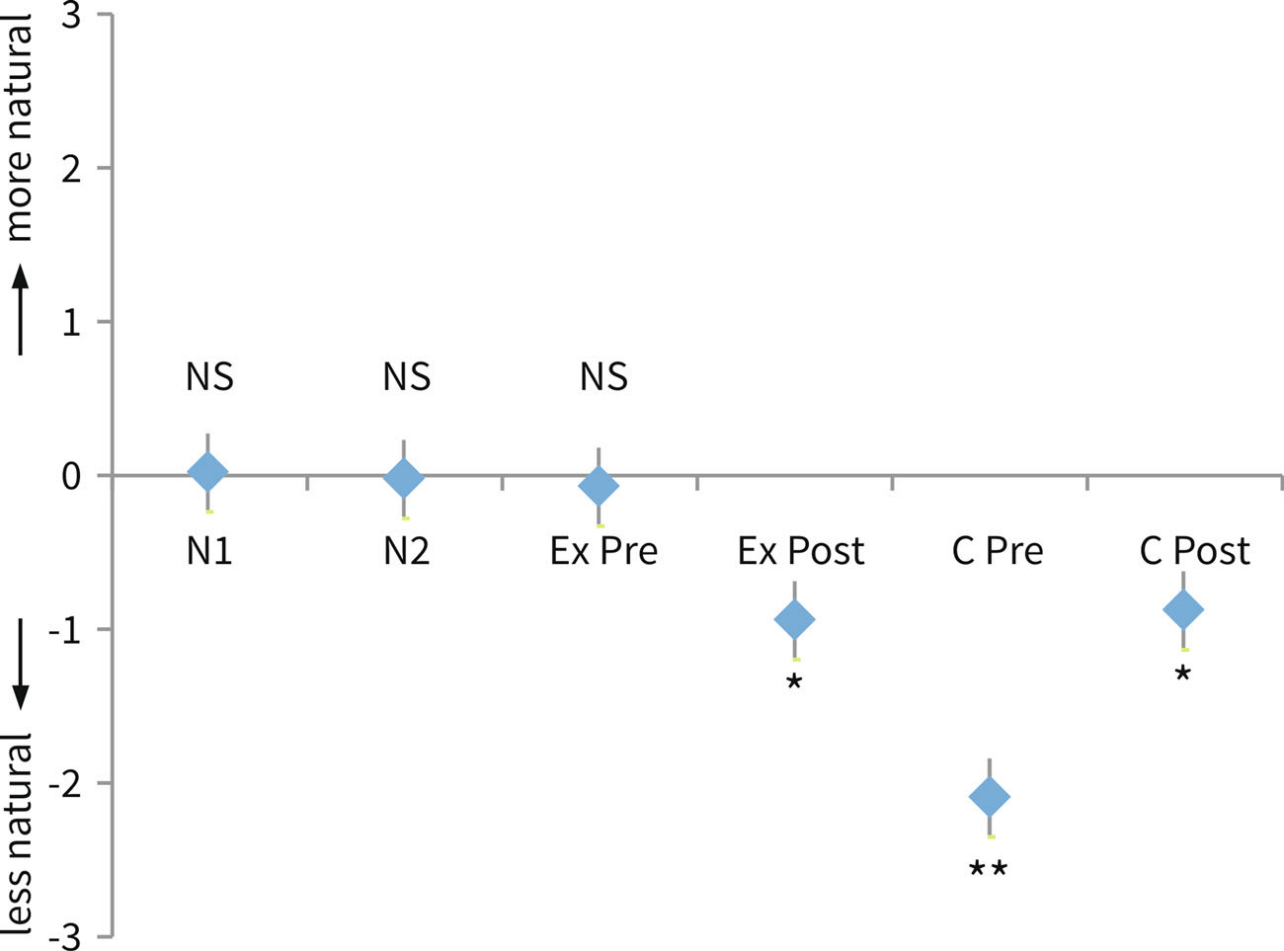
Results of the paired comparison ratings. Scores range from 3 to −3 on a seven point scale. A score of 3 means that the simulated ear canal acoustic c.q. filtered signal sounds much more natural than the reference c.q. unfiltered signal. A score of −3 denotes a clear preference in naturalness for the unfiltered signal. A score of 0 means that there is no noticeable difference in naturalness between the two signals. *Bars* denote de 95 % confidence interval for the mean. *Asterisk* significant difference with ‘normal’ conditions and Expre condition (*p* < 0.01), *double asterisk* significant difference with all other conditions (*p* < 0.01)

The ANOVA (mixed model) analysis showed no significant effect of the four different sentences used in the experiment. A second ANOVA analysis, with the gender of the speaker as a fixed effect, showed a small but significant effect of gender on perceived sound quality (*p* = 0.028). The mean rating score was 0.17 less natural for the male speaker, as opposed to the female speaker. More importantly, the different conditions significantly influenced outcome as can be seen in Fig. 2. When comparing the various conditions pairwise with the reference condition, there was no significant difference in naturalness between the two normal conditions (N1 and N2) and the pre-op exostosis condition (EXpre), since their rating scores were not significantly different from 0. In a pairwise comparison, all other conditions were perceived as significantly less natural (all *p* < 0.001). The pre-op cavity condition (Cpre) scored significantly less natural than all other conditions (all *p* < 0.001).

### VAS-scores

**Fig. 3:**
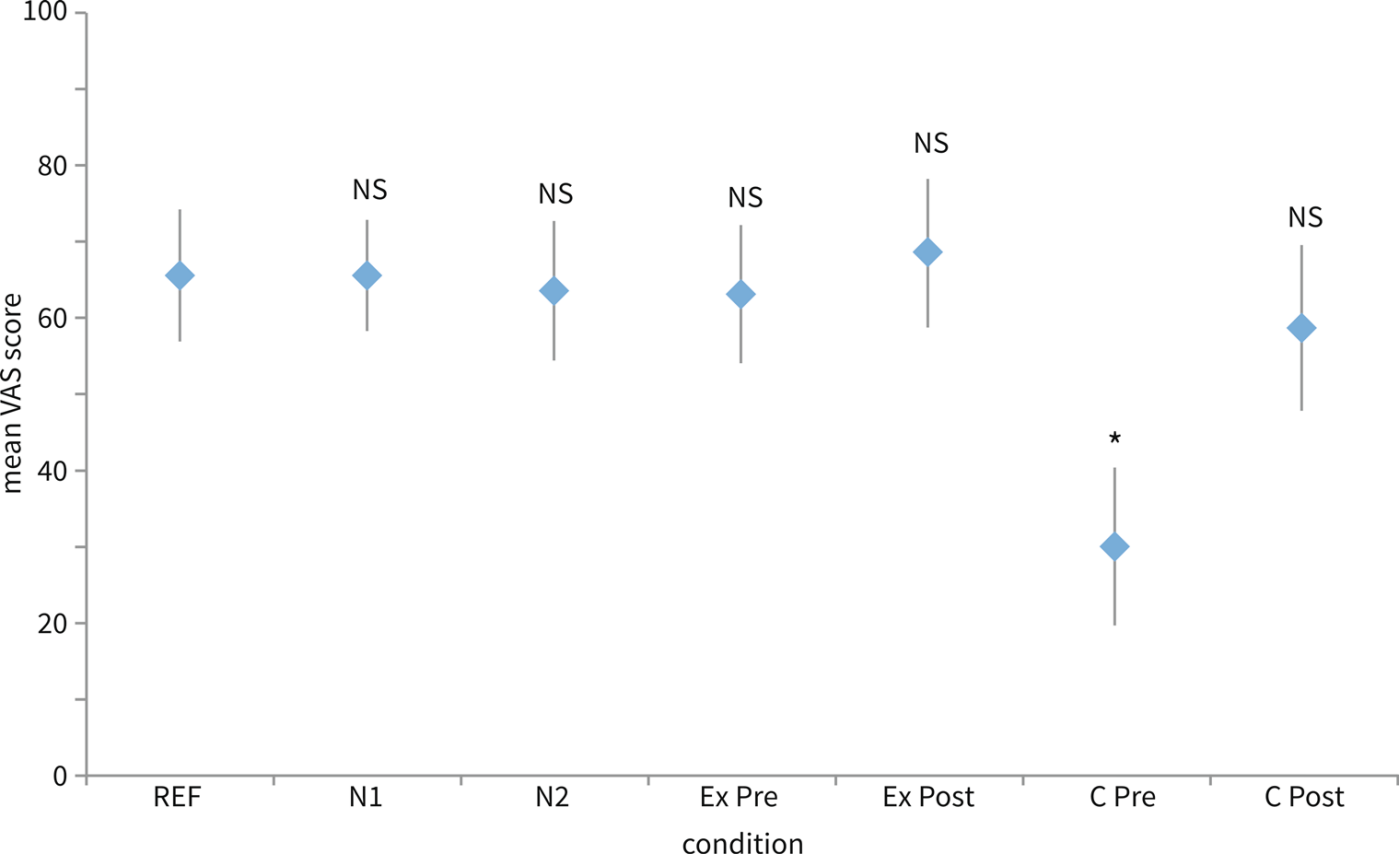
VAS evaluation of the percepted quality of the presented sound per condition. *REF* reference, *N1-2* ‘normal ear conditions’, *Ex pre and Ex post* exostosis condition pre and postoperative, *Cpre and Cpost* cavity condition pre- and postoperative, *NS* not significant compared to reference, *asterisk* significant difference with reference and other conditions (*p* < 0.01). *Bars* denote de 95 % confidence interval for the mean

The seven conditions presented are depicted in Fig. 3. No significant difference in VAS scores was observed between the reference condition and all other conditions (all *p* > 0.1), except the pre-operative radical cavity condition (*p* < 0.01).

## Discussion

Our data show that surgery on the osseous external auditory canal may result in clinically relevant changes in sound quality. This is in agreement with our prior work showing that extensive alterations have distinct and clear effects. The current study shows that less extensive changes also lead to distortions, albeit more subtle. This study clearly is an expansion of our earlier work as we used alterations of the osseous external auditory canal within one individual patient instead of multiple individuals. If one evaluates changes within one individual, possible effects of the meatal entrance on resonance function are eliminated. As shown in the present study, a canalplasty can result in a small deterioration in perceived quality of sound whereas a revision radical cavity with reconstruction of the posterior wall can result in a significant improvement. This suggests a volume related change. We hypothesize that a greater ear canal volume results in a deterioration of perceived sound quality. Yet it seems unlikely that ear canal volume is the sole contributor to this effect as both postoperative conditions were quite comparable, while the REURs differed considerably. It would seem that the REUR is also affected by the material of which the external auditory canal is constructed. As the field of otology is moving towards advocating obliteration techniques it is very interesting to determine how critical resonance effects are dependent on the material used for reconstructing the external auditory canal. Satar [11] has shown that obliterated cavities can achieve near normal resonance frequencies if residual volumes were in the normal range. As we still observed some difference in the resonance frequency in obliterated cavities, this may be explained by the materials used for reconstruction. Also, it could be that the normal mastoid cavity with its air cell tracts has a function in resonance, yielding a difference in resonance after obliteration of the entire mastoid cavity. This would be a novel function of the mastoid cavity as its aeration could be a part of how our hearing is influenced. Future studies are necessary to confirm this hypothesis.

We were surprised to find that a small, but significant, effect was found between male and female speech and we did not find a clear explanation. This finding suggests that spectral differences between male and female speech may have their effect on the sensitivity to perceive relatively subtle changes between conditions.

Although a significant change was observed between the reference and the post-operative conditions using the paired comparison technique this did not result in different VAS scores. Obviously, it is more difficult to determine the effect of the change without a clear comparison. This indicates that in future research both methods should be used as slight differences are found when comparison techniques are used. In unilateral cases this would be a comparison between the affected and non-affected ear if hearing levels in the audiogram would be preserved. Still, overall quality is a very subjective measurement and made without comparison and therefore it may well be that the VAS is a better representative for everyday functioning of the patient.

As sound quality can be divided into many subcategories (i.e. loudness, sharpness), one could debate whether our approach is too simple to evaluate the entire scope of ‘quality’ of perceived sound. We still feel that both tests provide valid and relevant data as these patient reported outcomes reflect daily practice. Therefore, they can be regarded as the primary outcome of a surgical intervention.

Using normal hearing subjects does give rise to some points of discussion. We know that the alteration of the acoustics of the external auditory canal is not the only effect surgery will elicit. Two other mechanisms suggested by Evans [5] are eliminated in our study design. First a conductive hearing loss of varying degree for different frequencies will be present, influencing overall sound quality. Yet our study design was aimed at exploring the isolated effect of the change in external ear acoustics on sound quality. Second, the change in middle ear volume in a canal wall down procedure and the type of tympanoplasty performed may also play an important role [2, 12, 13].

As our study did not consider these possible mechanisms for the abovementioned reasons, the study should be regarded as explorative and its usefulness in clinical practice remains to be proven. Other effects (for instance habituation and the interaction with the post-surgical hearing levels and hearing rehabilitation) have not been investigated and are open for future work. However, this study has strengthened our hypothesis that surgical procedures that alter the shape of the OEAC do effect overall quality of perceived sound. This would explain our clinical observations that some patients claim to have better or worse hearing without any change in post-operative audiogram. Also this effect should be considered in post-operative hearing aid fitting if hearing rehabilitation is (still) needed.

## Conclusion

This explorative study shows that commonly performed surgical procedures changing the shape of the OEAC do affect the resonance function and the perception of sound quality. These results seem to be influenced primarily by volume changes. In this study a canalplasty led to a small deterioration and an obliteration of a mastoid bowl in revision modified radical cavity surgery led to a significant improvement of perceived sound quality. To which extent these changes are important clinically (either in pre-operative counselling or post-operative hearing rehabilitation) remains to be determined.

## Electronic supplementary material

Below is the link to the electronic supplementary material.

Supplementary material 1 (WAV 305 kb)

Supplementary material 2 (WAV 305 kb)

Supplementary material 3 (WAV 305 kb)

Supplementary material 4 (WAV 305 kb)

Supplementary material 5 (WAV 305 kb)

Supplementary material 6 (WAV 305 kb)

Supplementary material 7 (WAV 305 kb)

## Abbreviations

OEAC: Osseous external auditory canal
VAS: Visual analogue scale
REUR: Real ear unaided response
REUG: Real ear unaided gain
PTA: Pure tone audiometry
